# Hepatitis C Virus Capsid Protein and Intracellular Lipids Interplay and its Association With Hepatic Steatosis

**DOI:** 10.5812/hepatmon.17812

**Published:** 2014-08-11

**Authors:** Muhammad Sohail Afzal, Najam Us Sahar Sadaf Zaidi, Jean Dubuisson, Yves Rouille

**Affiliations:** 1 Center for Infection and Immunity of Lille (CIIL), Institut Pasteur de Lille, Univ Lille Nord de France, Lille, France; 2 Atta ur Rahman School of Applied Biosciences, National University of Sciences and Technology, H-12 Islamabad, Pakistan

**Keywords:** Hepatitis C Virus, Fatty Liver, Intracellular Lipids, Steatosis

## Abstract

**Background:**

Hepatitis C Virus (HCV) is a major causative agent for chronic liver disease worldwide. Hepatic steatosis is a frequent histological feature in patients with chronic HCV. Both host and viral factors are involved in steatosis development. It results from uncontrolled growth of cytoplasmic lipid droplets (LDs) in hepatocytes. LDs are intracellular organelles playing key role in the HCV life cycle. HCV core protein localizes at the LD surface and this localization is crucial for virion production.

**Objectives:**

We explored in vitro interplay of core and LDs to investigate the role of core in steatosis.

**Materials and Methods:**

Core expression vectors were transfected in Huh-7 cells. The effect of core protein on LDs content and distribution in the cells was monitored by confocal microscopy. Cells were treated with oleic acid to analyze the effect of increased intracellular LDs on core expression. Core protein expression was monitored by western blot analysis.

**Results:**

Core expression altered the intracellular lipid metabolism, which resulted in a change in LDs morphology. Core LDs interaction was required for this effect since the mutation of two prolines (P138A, P143A), which impair LDs localization, had no impact on LDs morphology. Conversely, oleic acid induced intracellular LD content resulted in increased core expression.

**Conclusions:**

Core-LDs interaction may be an underlying molecular mechanism to induce liver steatosis in patients with HCV infection. This interaction is also crucial for efficient viral replication and persistence in infected cells. Steatosis can also interfere with efficient standard interferon therapy treatment. Management of steatosis should be considered along with standard care for achieving higher sustained virological response (SVR) in patients receiving interferon regimen.

## 1. Background

Fatty liver or hepatic steatosis is defined as excessive lipid accumulation in hepatocytes cytoplasm. It is a common finding in the general population and is a frequent cause of elevated serum amino-transferase levels (1). Generally, hepatitis C virus (HCV) infection and hepatic steatosis are independent. However the actual co-occurrence of both pathologies is much higher than chance (2). This suggests an association between these two independent diseases (3). Severity of steatosis correlates with the level of HCV replication in liver (4) or in serum (5) of infected individuals and is significantly reduced or even disappears after successful treatment of patients with standard antiviral therapy. There is little information regarding the mechanisms leading to lipid accumulation in infected hepatocytes. However, there is strong evidence to suggest that some HCV proteins, particularly the structural capsid protein and the nonstructural protein, NS5A, can induce hepatic steatosis (reviewed in (6)). HCV uses intracellular lipid droplets (LDs) for successful completion of infectious viral life cycle. HCV capsid protein localizes on LDs (7-9) and this LD-associated core protein recruits viral replication complexes to lipid droplet as a first step of virion assembly (10). HCV core protein is a 191-residue long, highly basic, RNA binding protein. It consists of three domains namely D1, D2 and D3. D1 is a hydrophilic N-terminal domain of 117 residues, D2 a hydrophobic domain located between residues 117 and 169, and D3 a highly hydrophobic domain of 22 residues, which serves as the signal peptide of the E1 protein located downstream in the precursor (8). Signal peptidase-mediated cleavage of the polyprotein generates an immature form of core protein (p23), which undergoes further cleavage by the intramembrane-cleaving protease signal peptide peptidase (SPP), leading to the removal of most of domain D3 and the production of mature p21 capsid protein (11). The mature form of core is a membrane-associated dimeric α helical protein (8). The SPP-mediated cleavage is essential for core localization at the LD surface.

## 2. Objectives

The aims of this study were to evaluate the role of HCV capsid protein and intracellular LD interaction on LDs content and whether this interaction has any impact on core protein expression.

## 3. Materials and Methods

The study was conducted at the "Molecular and Cellular Virology of Hepatitis C lab, Institut Pasteur de Lille, France" in 2011-2012.

### 3.1. Chemicals, Antibodies and Plasmids

Dulbecco's modified Eagle's medium (DMEM), goat and fetal calf sera (FCS), phosphate-buffered saline (PBS), Paraformaldehyde (PFA), gentamicin, 4',6-diamidino-2-phenylindole (DAPI), and BODIPY 493/503 (Life Technologies, France), Oleic acid (Sigma, France), Mowiol (Calbiochem, France). Mouse monoclonal anti-core antibody ACAP27 (BioRad, France), Cy3-conjugated goat anti-mouse IgG, and HRP-conjugated secondary antibody (Jackson Immunoresearch, West Grove, PA, USA), Mouse monoclonal anti-tubulin antibody (Sigma, France) were obtained. Core coding sequence was amplified by PCR from the plasmid pJHF1-CSN6A4 (12) and subcloned into the expression vector pCI-neo (Promega, France) between a Kozak consensus sequence and a stop codon. Core protein PP mutant expression vector was produced by replacing the codons of residues P138 and P143 with alanine codons by overlapping PCR. Constructs were confirmed by DNA sequencing.

### 3.2. Cell Culture

Human hepatoma-derived Huh-7 cells (13) were grown in DMEM supplemented with 10% FCS and 10 μg/mL gentamicin.

### 3.3. Transfection

Transfection was performed as described previously (14). Huh-7 cells were grown on glass cover slips approximately 24 hours prior to transfection. The transfection was performed using the following protocol: 25 μL of serum-free medium was mixed with 2 μL of transfection reagent TransIt-LT1 (Mirus Bio, USA) and incubated at room temperature for five minutes. 0.5 μg of core expression plasmid was added and the mixture was further incubated for 30 minutes. The transfection mixture was then added drop-wise to freshly added cell culture medium (0.5 mL), mixed and incubated at 37 ^°C^.

### 3.4. Immunofluorescence Microscopy

Indirect immunofluorescent labeling was performed as previously described (14). Huh-7 cells grown on cover slips were washed with PBS and fixed with 3% PFA for 20 minutes. Cells were then permeabilized for three minutes with 0.1% triton X-100, blocked with 10% goat serum, and labeled for 30 minutes with core-specific mouse monoclonal antibody. After PBS washes, cells were incubated with Cy3-conjugated goat anti-mouse IgG antibody and DAPI to label nuclei. Cells were incubated for 10 minutes with 0.2 μg/mL BODIPY 493/503 to stain LDs. The coverslips were then mounted on slides by using Mowiol-containing mounting medium. Images were obtained with an LSM710 laser-scanning confocal microscope (Zeiss, Germany) using a 63X/1.4 numerical aperture oil immersion objective. Signals were sequentially collected using single fluorescence excitation and acquisition settings to avoid crossover. Images were processed using Adobe Photoshop software (Adobe, France).

### 3.5. Immunoblotting

Immunoblotting was performed as described previously (14). Briefly, cells were lysed in a solution containing 50 mM Tris-Cl buffer, pH 7.5, 100 mM NaCl, 1 mM EDTA, 1% Triton X-100, 0.1% sodium dodecyl sulfate (SDS), and protease inhibitors (Sigma-Aldrich, France), for 30 minutes on ice. Cell lysates were precleared by centrifugation. Protein concentration was determined in the postnuclear supernatants by the BCA method using bovine serum albumin as a standard. The proteins were resolved by SDS–12.5% PAGE and transferred onto nitrocellulose membranes (Hybond-ECL; Amersham, USA) using a Trans-Blot apparatus (Bio-Rad, France). Proteins of interest were revealed with specific primary antibodies, followed by species-specific secondary antibodies conjugated to peroxidase. Enhanced chemiluminescence solution (ECL Plus, GE healthcare, USA) was used to visualize proteins. The signals were recorded using LAS 3000 (Fujifilm, USA), and quantified with ImageJ.

### 3.6. Statistical Analysis

All experiments were performed in duplicate wells. All experiments were repeated three times with consistent results. The mean and standard error (SE) were calculated using MS excel.

## 4. Results

**Figure 1.**
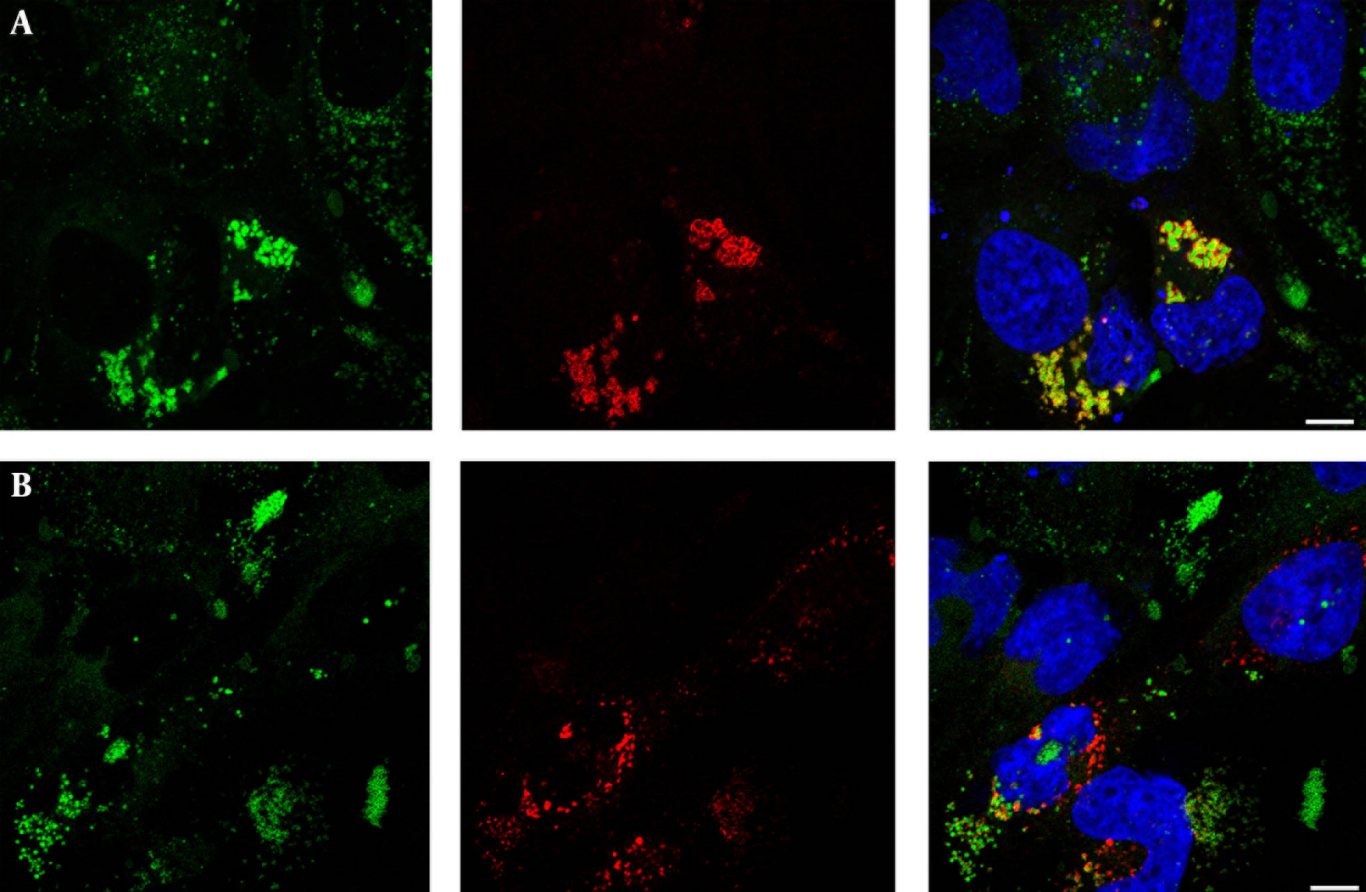
Impact of Core Expression on LD Morphology Huh-7 cells were transfected with plasmids expressing wild type core (A), or core PP mutant (B). Cells were fixed at 24 hours after transfection, and core protein was detected with MAb anti-core ACAP-27 (red). Lipid droplets were stained with Bodipy 493/503 (green). Merged images are shown, with nuclei stained with DAPI (blue). Representative confocal images are shown.

To assess the role of core on LDs morphology, we transfected Huh-7 cells with expression vectors for wild-type core or a mutant form of core not targeted to LDs. Cells were double-labeled to detect core expression and LDs. In wild-type core-expressing cells, core co-localized with LDs (Figure 1 A). In these cells, LDs were larger and brighter than cells with no core expression, indicating an increased intracellular LD content in core-expressing cells (Figure 1 A). To evaluate whether this increase of intracellular LD content is due to core localization on LD, cells were transfected with a core PP mutant not targeted to LDs. As expected, no co-localization of core with LD was observed (Figure 1 B). In contrast to wild-type core expressing cells, the intracellular LD content was similar in cells expressing core PP mutant and in untransfected cells (Figure 1 B).

**Figure 2.**
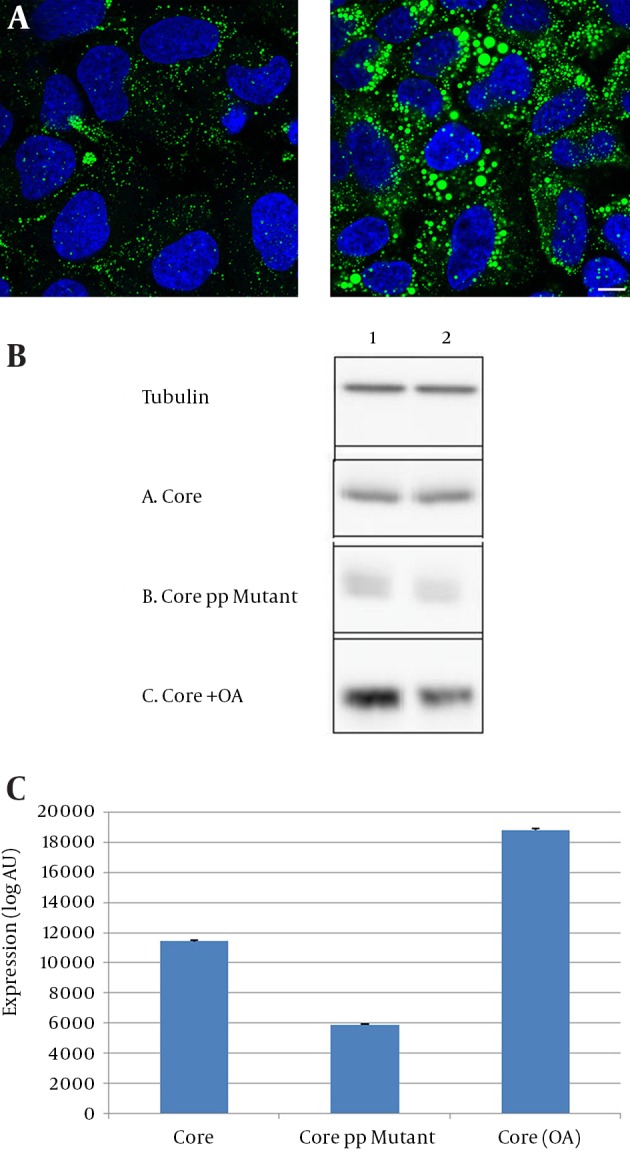
Impact of LDs on Core Protein Expression (A) Huh-7 cells grown on glass cover slip were cultured for 16 hours in the presence of 100 µM oleic acid (right panel) or in control condition (left panel). Cells were fixed and labeled with Bodipy 493/503 to detect cytoplasmic lipid droplets (green) and with DAPI to detect nuclei (blue). Representative confocal images are shown. (B) Huh-7 cells were transfected with plasmids expressing wild type core, or core PP mutant or core in OA treated cells. Cells were lysed after 40 hours of transfection. Core and Tubulin were revealed by immunoblotting. Each treatment was performed on two samples of cells. (C) Core protein bands were quantified by using ImageJ software and the graph showed core protein expression. The graph showed the mean ± SE of three independent experiments.

To check the effect of increased intracellular LDs on core protein expression, transfected Huh-7 cells were treated with 100 µM oleic acid (OA). As expected, OA treatment increased the size and number of intracellular LDs (Figure 2 A). The expression levels of core were assessed by immunoblotting. Core expression was more prominent in OA-treated cells than untreated cells (Figure 2 B). Accordingly, core expression was lower in core PP mutant transfected cells, showing the importance of LDs in core expression (Figure 2 B, 2 C).

Core expression also changed the distribution pattern of LDs. In cells expressing the wild type core, LDs were located around nucleus while in cells with no core expression LDs were scattered throughout the whole cytoplasm of the cell (Figure 1 A). The cells expressing core PP mutant also had LDs scattered all over the cytoplasm (Figure 1 B), indicating that the perinuclear LDs distribution required core to be targeted to LDs.

## 5. Discussion

In patients with HCV infection, hepatocellular steatosis is a cofactor for fibrosis, disease progression and chronicity (5). In steatosed hepatocytes, alteration of lipid metabolism results in the accumulation of larger LDs. Here we showed that HCV capsid protein alone is sufficient to increase the intracellular LD content. Core expressing cells showed higher amounts of intracellular lipids and this increased lipid content was due to core localization on LD surface. The cells expressing a mutant core protein not targeted to LDs (PP mutant) had lower amounts of intracellular lipids, evident that core localization on LD surface is required for the accumulation of intracellular LDs. Moreover, when core was expressed in cells with LDs in increased number and size, a state similar to liver steatosis, core expression was higher than normal cells. Core expression appears to increase the intracellular LD content, and an increased LD content in turn increases core expression, a state mimicking the favorable condition for HCV replication. HCV may interfere with lipid metabolism and results in steatosis by utilizing at least three distinct, non-mutually exclusive mechanisms; impaired secretion, increased de novo synthesis, and impaired degradation (15). The results of the current study showed that in core expressing cells LDs were located in a perinuclear region, while in PP mutant core expressing cells LDs were distributed in the whole cytoplasm, just like in naive cells. The intracellular LD content was also higher in core expressing cells as compared to uninfected or pp mutant core expressing cells. This indicates that core protein is responsible for redistribution and enhanced accumulation of LDs, which was shown to be associated with efficient virion production and blocking LDs redistribution through microtubule network resulted in reduced release of infectious progeny (16). The expression of HCV replicon lacking the core protein resulted in no enhancement of LDs in cultured Huh-7 cells (10) indicating that expression of this protein alone caused LD accumulation. HCV core interferes with lipid synthesis machinery through a number of unrelated mechanisms, which involve host factors like apolipoprotein B (ApoB), cholesterol, very-low density lipoprotein (VLDL) assembly and/or secretion, microsomal triglyceride transfer protein (MTP) activity, sterol regulatory element binding protein (SREBP)-1c signaling pathway, peroxisome proliferators-activated receptor (PPAR)-α (reviewed in (15)). Our results showed that HCV hijacks lipid biosynthesis and degradation pathways and uses it for its own benefits. Core protein interferes with lipid degradation pathway and resulted in increased intracellular LDs. Core protein expression was higher in cells with increased LDs compared to cells having low intracellular LDs. Increased intracellular LDs content provides favorable environment for efficient viral replication, which supported the fact that HCV does not have a latent phase (17). Throughout the course of the infection, which can be decades long in many patients, there is constant production of viral RNA, proteins and infectious particles. Increased LDs content and efficient viral replication could be one of the possible mechanisms for viral persistence in chronic patients. LDs and LD-associated ER-membranes supply lipids for nascent HCV to produce efficient infectious HCV virion (18, 19). Core protein localized on LDs recruits the replication complexes to the LD-associated membranes via the core-NS5A interaction for virus assembly (20, 21). Disruption of core-LD association impaired infectious viral particles production (10). LD-dependent HCV production pathway may be an important component of future strategies for the prevention and treatment of HCV infection. The limitation of the study was that we did not use full-length HCV cell culture model to confirm that increased core expression mimics increased HCV virion production. Persistent HCV infection depends on both host and viral factors (22-27). Viral genotype (22, 23), viral immune escape mutations, adaptations (23, 24), host genetic factors (25, 26), and HCV specific INF-γ responses (27) are important in patients' prognosis. HCV is endemic in Pakistan, with more than 10 million infected individuals (28). The common prevalent genotype is 3a (29), which is worse than all other viral genotypes regarding steatosis development (4). In patients with genotype 3 infection, steatosis severity is directly linked to viral load (4, 5) and is significantly reduced or even disappears when patients are successfully treated (5, 30). In spite of the fact that genotype 3a showed very high-sustained virological response (SVR) for interferon treatment, HCV burden is rising in Pakistan. This might be linked to higher steatosis in infected patients, which results in lower SVR. Hepatic steatosis could be a result of viral, host genetic and dietary factors. Management of steatosis should be considered when treating HCV patients with standard interferon treatment.
